# 188 Recovery after a sports-related concussion: a longitudinal study of adolescent rugby union players in Northern Ireland

**DOI:** 10.1093/eurpub/ckae114.106

**Published:** 2024-09-26

**Authors:** Connor McKee, Mark Matthews, Alan Rankin, Chris Bleakley

**Affiliations:** Ulster University, Belfast, United Kingdom; Ulster University, Belfast, United Kingdom; Sports Institute of Northern Ireland, Belfast, Northern Ireland; Sports Medicine NI LTD, Belfast, Northern Ireland; Ulster University, Belfast, United Kingdom

## Abstract

**Purpose:**

Adolescent athletes who sustain a sports-related concussion may experience a prolonged recovery period. Evidence suggests female athletes and those with a history of previous concussion may have an extended recovery period, spanning multiple weeks to months. The purpose of this study was to track recovery from a concussion across patient-reported measures and determine the time taken to return to pre-injury levels in adolescent rugby union players.

**Study design:**

A longitudinal study was utilised across a single rugby union playing season (2022-23). Ethical approval was granted from Ulster University Research Ethics Committee

**Methods:**

Male and female rugby union players were recruited from nine school and club rugby teams across Northern Ireland. To be eligible, participants had to be 16-18years of age, injury free and currently playing at First XV level. Participants completed demographic and established questionnaires including Post-Concussion Symptom Scale (PCSS), Concussion Clinical Profiling (CP), Paediatric Fear Avoidance Behaviour after Traumatic Brain Injury (PFAB-TBI), General Anxiety Disorder (GAD) and Patient Health Questionnaire (PHQ). Those who sustained a concussion were re-assessed at 3, 7, 14, 23, 90 and 180days post-event. Recovery was defined as questionnaire score at pre-injury level. The primary outcome measure was Post-Concussion Symptom Scale.

**Results:**

Of the 149 participants (113M (76%); 36F (24%)), 11 (7%) sustained a concussion during the season (9M: 2F), of which four had a previous history of concussion (2M: 2F). PCSS and PFAB-TBI took the longest time to return to baseline scores. Statistically significant differences in survival distribution (Chi-square 9.27 (df = 4) p < 0.05) across self-reported outcomes; pairwise comparisons show the largest differences in survival distribution were seen between PCSS and GAD (p = 0.02) and PCSS and PHQ (p < 0.04).

**Conclusions:**

Adolescent male and female rugby union players experienced prolonged post-concussive symptoms based on self-reported measures. Further research on male and female adolescent athletes is needed to track recovery across various clinical measures.

**Funding:**

This study was funded by the Department for the Economy (DfE) as part of a sponsorship of postgraduate students

